# UGT1A6 Polymorphisms Modulated Lung Cancer Risk in a Chinese Population

**DOI:** 10.1371/journal.pone.0042873

**Published:** 2012-08-17

**Authors:** Ley-Fang Kua, Soo Ross, Soo-Chin Lee, Kousaku Mimura, Koji Kono, Boon-Cher Goh, Wei-Peng Yong

**Affiliations:** 1 Department of Haematology-Oncology, National University Cancer Institute of Singapore, Singapore, Singapore; 2 Cancer Science Institute of Singapore, National University of Singapore, Singapore, Singapore; 3 Department of Sugery, Yong Loo Lin School of Medicine, National University of Singapore, Singapore, Singapore; IPO, Inst Port Oncology, Portugal

## Abstract

Uridine diphosphoglucuronosyltransferases (UGTs) 1A6 is the only UGT1A isoform expressed in lung tissue. It is responsible for the detoxification of carcinogens such as benezo[a]pyrene from cigarette smoke. The purpose of this study was to evaluate the association of UGT1A6 polymorphisms and haplotypes with lung cancer risk and to evaluate the functional significance of UGT1A6 polymorphisms. Genomic DNA was isolated from leukocytes. Eight UGT1A6 polymorphisms were sequenced in a test set of 72 Chinese lung cancer patients and 62 healthy controls. Potential risk modifying alleles were validated in a separate set of 95 Chinese lung cancer patients and 100 healthy controls. UGT1A6 19T>G, 541A>G and 552A>C showed significant association with increased lung cancer risk, while UGT1A6 105C>T and IVS1+130G>T were significantly associated with reduced lung cancer risk. Multivariate logistic regression analysis demonstrated a significant association of lung cancer with UGT1A6 541A>G (OR: 3.582, 95% CI: 1.27–10.04, p = 0.015), 552A>C (OR: 5.364, 95% CI: 1.92–14.96, p = 0.001) and IVS1+130G>T (OR: 0.191, 95% CI: 0.09–0.36, p<0.001). Functional test demonstrated that UGT1A6 105C>T increased mRNA stability, providing a plausible explanation of its association with reduced lung cancer risk. Thus UGT1A6 polymorphisms may be used to identify people with increased risk of developing lung cancer.

## Introduction

Non-small cell lung cancer (NSCLC) accounts for more than 85% of primary lung cancers and approximately two-thirds of NSCLC patients are diagnosed at an advanced stage. Cigarette smoking has been linked to lung cancer [1]–[4], and over 60 carcinogens had been identified in cigarette smoke [5]–[7]. Of these, benzo[a]pyrene (BaP) and 4-(Methylnitrosamino)-1-(3-pyridyl)-1-butanone (NNK) are the most studied. Metabolic activation of BaP and NNK by cytochrome P450 enzymes results in the formation of more reactive metabolites that can bind covalently to DNA, an important step in lung cancer induction [8]–[10].

The uridine diphosphoglucuronosyltransferases (UGTs) belong to a superfamily of metabolizing enzymes responsible for detoxifying numerous endobiotics and xenobiotics [11]. UGTs are responsible for the detoxification of BaP and NNK [12]–[15]. Lower glucuronidation activity had been implicated in lung cancer susceptibility [16]. In addition, genetic polymorphisms in UGTs have correlated with risk and incidence of different cancers including lung cancer [12], [17]–[20]. Amongst all UGT1A isoforms, UGT1A6 had been shown to be the only UGT1A isoform expressed in lung tissue [21].

We hypothesized that polymorphisms in the UGT1A6 may affect the ability to detoxify lung cancer carcinogens and modulate lung cancer risk. Here, we present the findings of a case-control study using 2 different cohorts and functionally characterized UGT1A6 105C>T polymorphism *in vitro*.

## Materials and Methods

### Study population

A total of 167 Chinese lung cancer cases and 162 Chinese healthy controls from 2 independent cohorts were analyzed. The first cohort comprised of 72 lung cancer patients and 62 healthy controls from the Cancer Center and Blood Donation Center from the National University Hospital, Singapore, respectively. A second cohort consisted of 95 lung cancer patients and 100 controls from 4 clinical studies and was used as validation set. The subjects in this cohort were lung cancer patients from two therapeutic phase II studies (n = 44 and 14 respectively) and a germline biomarkers study (n = 37) and eligible cancer-free controls from a case-control study of familial colorectal cancer in Singapore. The study protocol was approved by the institutional ethnics review board at the National University Hospital, Singapore, and all study subjects provided written informed consent.

### Data collection

In the first cohort, study subjects were interviewed in person by trained medical professionals who used a structured questionnaire. Demographic information including age, gender, smoking status, pack years of smoking and histological cell type was collected for all subjects. Similar information for the second cohort was retrieved from case records, and information on the smoking status for the controls was not obtained. Smoking status was categorized into never-smoker, ex-smoker and current smoker. Histological cell types were categorized into three groups, namely adenocarcinoma, squamous cell carcinoma and poorly differentiated carcinoma.

### Single Nucleotide Polymorphism (SNP) genotyping

Eight SNPs which were relatively common in the Asian population based on past literature search, were selected for this study. Of the four variants in exon 1, three were coding (cSNPs) resulting in amino acid changes, UGT1A6 19T>G (S7A), 541A>G (T181A) and 552A>C (R184S), whereas UGT1A6 105C>T was a synonymous variant. The remaining three sequence variants in the 5′ regulatory region included 2 SNPs, UGT1A6 −556C>T and −427G>C, and one deletion mutation −1310 to −1306 delete (aggag). UGT1A6 intronic variant IVS1+130G>T was also studied.

Genomic DeoxyriboNucleic Acid (DNA) was extracted from buffy coat fractions using Puregene DNA purification kit (Gentra Systems, Inc., Minneapolis, MN, US). The quality and quantity of DNA were assessed by NanoDrop (ND-1000 Spectrophotometer). All DNA has an A_260_/A_280_ ratio of 1.7–1.9. Genotyping was conducted using Pyrosequencing. For pyrosequencing assays, one positive and one negative control were included in each 96-well plate. Briefly, primers were designed based on the UGT1A6 sequence. The PCR primers and sequencing primers were designed using Pyrosequencing Assay Design Software (version 1.0.6: Biotage AB, Uppsala, Sweden). BLAST analysis was performed to confirm the specificity of the primers for each exon. Details of primer sequences and the PCR conditions are listed in Table S1. 250 ng genomic DNA at 50 ng/µl was used for each PCR reaction and PCR products were then subjected to pyrosequencing with PyroMark™ ID device (Pyrosequencing, Uppsala, Sweden). Validation steps were carried out by direct sequencing reactions using the ABI PRISM® BigDye™ terminator v 3.1 cycle sequencing chemistry.

### Plasmid constructs

Functional studies on nonsynonymous polymorphisms (UGT1A6 19T>G, 541A>G and 552A>C) have been done, whereas IVS1+130G>T is an intronic SNP, therefore we selected UGT1A6 105C>T, which is a synonymous polymorphism for our functional testing. To evaluate whether UGT1A6 105C>T has any impact on UGT1A6 activity, the variant 105TT was generated. Briefly, plasmid vector encoding full-length UGT1A6*1 sequence was kindly provided by Dr. Michael H. Court (Tufts University School of Medicine, Boston, Massachusetts). The UGT1A6 coding region was subcloned into pcDNA 3.1 V5/His Topo expression plasmid vector (Invitrogen). BLAST analysis was performed to confirm the UGT1A6 sequence of the plasmid. The plasmid was then used as template to generate substitution at nucleotide 105 by using the GeneEditor *in vitro* site-directed mutagenesis system (Promega) with the indicated primer 105CT (5′-5Phos/ccc tca gga Tgg aag cca c-3′) and primer Bottom Strand (provided). All DNA constructs were verified by sequencing analysis. Briefly, HEK293 cells were cultured in Dulbecco's modified Eagle's medium supplemented with 10% fetal bovine serum and 50 U/ml penicillin (Gibco) in a humidified atmosphere of 5% carbon dioxide at 37C. Stable transfections of plasmid into HEK293 cells were performed using Lipofectamine 2000 (Invitrogen) at a reagent to DNA ratio of 3∶1 in Opti-MEM® I Reduced Serum Medium (Gibco). All transfection experiments were carried out in triplicates.

### RNA stability assay

We next examined the possible effect of the variant UGT1A6 105TT on mRNA stability. 10 µg/ml actinomycin D (Sigma) was applied to the cell cultures grown overnight and cells were harvested at selected intervals up to 24 hours. The Rneasy Plus Mini kit (Qiagen) was used to extract total RNA from transfected cells, before and after incubation with actinomycin D.

### Quantification of transcripts

RNA was isolated from 5×10^5^ cells at the indicated times (0, 2, 4, 8 and 24 hours) after treatment with ActD and were reverse transcribed to cDNA using SuperScript III (Invitrogen). The PCR reaction was then carried out comprising the following: 1 µl cDNA, 10 µM of each primers (UGT1A6: 5′-cagctgtcctcaagagagatgtgga-3′ and 5′-ccaatgaagaccatgttgggc-3′, GAPDH: 5′-tgaaggtcggagtcaacggatttggt-3′ and 5′-catgtgggccatgaggtccaccac-3′) and 2× Power SYBR Green Master Mix (which include SYBR Green dye, Taq Polymearse, ROX, and dNTP) in a final volume of 20 µl. Empty vector-transfected cells was utilized as control, with data normalized by GAPDH expression. All PCR reactions were carried out in triplicates.

### Western blotting

We further examined the effect of the variant UGT1A6 105TT on protein stability. Treated cells were lysed and protein concentrations were measured. Proteins (7.5 µg/well) were resolved by 4 to 15% gradient SDS-polyacrylamide gel electrophoresis (Invitrogen) followed by transferred to a polyvinylidene fluoride (PVDF) microporous membrane (MILLIPORE, Billerica, MA, USA) blocked in PBS with 5% milk. After blocking, the membrane was probed using a rabbit anti-human UGT1A6 primary antibody diluted 1∶1000 (WB-UGT1A6; BD Gentest, Woburn, MA) and a goat anti-rabbit horseradish peroxidase-conjugated secondary antibody (1∶10,000 diluted). Blots were imaged by ECL reagents (ECL Prime, Amersham Pharmacia Biotech AB, Uppsala, Sweden) and by film processor (Konica SRX 101). Results are from two independent experiments.

### Enzyme assay

Serotonin was used as substrate as it demonstrated substrate specificity to UGT1A6 [22], [23], however, a different platform methodology was used in this study. 0.01 mg of proteins was subjected to 384-well black plate (Corning, NY) with Transcreener ® HTS Fluorescent Intensity (FI) Assay kit (BellBrook Labs, Madison, WI). Each enzyme reaction was carried out comprising the following: 50 mM HEPES (pH 7.5), containing NaCl (100 mM), MgCl_2_ (4 mM), EGTA (2 mM), 0.01% Brij-35, dithiothreitol (DTT; 2 mM), DMSO (1%), UDPGA (5 mM) and with varying concentrations of serotonin (Sigma) 0.5–30 mM in a final volume of 25 µl. The plate was incubated at RT for 45 min and reactions were stopped by the addition of 25 µl of ice-cold methanol. The plate was then read on a Tecan Infinite M200 plate reader. All enzyme reactions were carried out in triplicates. The initial readouts were converted to the amount of UDP formed using GraphPad Prism to interpolate these values from the UDPGA/UDP standard curves. Michaelis-Menten curves were generated using GraphPad Prism. Data plotted are from two independent experiments and results are presented as the means ± S.D. of triplicates.

### Statistical Analyses

Case-control differences in baseline characteristics were evaluated using t-tests (for continuous variables) and Chi-square tests (for categorical variables). Odds ratios (OR) were derived from Chi-square test, and 95% confidence intervals (CI) of the OR were given. The association between SNPs with age, gender, smoking status and TNM stages were tested in lung cancer subjects using chi-square test. All statistical analysis including univariate and multivariate logistic regression analysis were performed using SPSS version 11.5 (SPSS Inc., Chicago,Ill.,USA). Hardy-Weineberg equilibrium test, linkage disequilibrium analysis (denoted as D′) and case-control haplotype analysis (permutation test) were calculated using SNPAlyze ver.7.

## Results

### Characteristics of patients and controls

Baseline characteristics of lung cancer patients and controls of both cohorts are summarized in Table 1. There was a statistically significant difference in age, gender and smoking status between lung cancer patients and healthy controls, with more male (p = 0.003) and smokers (p<0.001) in the lung cancer group. Compared to healthy controls, patients with lung cancers, were generally older (p = 0.04). Adenocarcinoma was the most common histological subtype, accounting for 60% in lung cancer patients. There was no significant difference in the baseline characteristics of lung cancer patients and healthy controls between the testing and validation cohorts (data not shown).

**Table 1:** Baseline characteristics of Lung cancer patients and controls.

Characteristics	Patients = 167 (%)	Controls = 162 (%)	p-values
Age *(Range)*	63 (39–79)	36 (18–67)	0.04
Gender			
Male	129 (77%)	100 (62%)	0.003
Female	38 (23%)	62 (38%)	
Smoking status			
Never	55 (33%)	120 (74%)	<0.001
Ex-smoker	55 (33%)	26 (16%)	
Current smoker	57 (34%)	16 (10%)	
Pack-years of smoking *(Range)*	21.6 (0–160)	0 (0–35)	ns
Histological Cell Type			
Adenocarcinoma	101 (60%)		
Squarmous cell carcinoma (SCC)	25 (15%)		
Poorly differentiated	41 (25%)		

*Smoking history was not obtained for the 100 controls.

### Genotype and allele frequencies of UGT1A6 polymorphisms (Table 2)

**Table 2:** Genotype and allele frequencies of *UGT1A6* SNPs in both first and second cohorts.

First cohort: 72 lung cancer patients and 62 controls							
SNPs	Genotyping frequency (%)			Allele frequency (%)		p-value	OR (95%CI)
−1310del5 (rs45549435)	Aggag/Aggag	Aggag/–	–/–	Aggag	–		
Patients	50 (69)	18 (25)	4 (6)	0.82	0.18	0.4903	0.783 (0.389 to 1.571)
Controls	38 (61)	22 (36)	2 (3)	0.79	0.21		
−556C>T (rs45568235)	CC	CT	TT	C	T		
Patients	70 (97)	2 (3)	0 (0)	0.99	0.01	0.5303	0.562 (0.091 to 3.478)
Controls	59 (95)	3 (5)	0 (0)	0.98	0.02		
−427G>C (rs12476197)	GG	GC	CC	G	C		
Patients	49 (68)	23 (32)	0 (0)	0.84	0.16	0.9691	0.986 (0.476 to 2.040)
Controls	45 (73)	16 (26)	1 (2)	0.86	0.15		
19T>G (rs6759892)	TT	TG	GG	T	G		
Patients	19 (26)	51 (71)	2 (3)	0.62	0.38	<0.0001	5.579 (2.681 to 11.61)
Controls	40 (65)	22 (35)	0 (0)	0.83	0.17		
105C>T (rs45535938)	CC	CT	TT	C	T		
Patients	55 (76)	17 (24)	0 (0)	0.88	0.12	0.0376	0.458 (0.217 to 0.963)
Controls	37 (60)	25 (40)	0 (0)	0.8	0.2		
541A>G (rs2070959)	AA	AG	GG	A	G		
Patients	7 (10)	65 (90)	0 (0)	0.55	0.45	<0.0001	25.07 (9.819 to 64.00)
Controls	48 (77)	14 (23)	0 (0)	0.89	0.11		
552A>C (rs1105879)	AA	AC	CC	A	C		
Patients	5 (7)	65 (90)	2 (3)	0.52	0.48	<0.0001	22.72 (7.992 to 64.60)
Controls	38 (61)	24 (39)	0 (0)	0.8	0.2		
IVS1+130G>T (rs7592281)	GG	GT	TT	G	T		
Patients	55 (76)	16 (23)	1 (1)	0.88	0.12	<0.0001	0.241 (0.116–0.502)
Controls	26 (43)	36 (57)	0 (0)	0.72	0.28		

Second cohort: 95 lung cancer patients and 100 controls							
SNPs	Genotyping frequency (%)			Allele frequency (%)		P-value	OR (95%CI)
19TG (rs6759892)	TT	TG	GG	T	G		
Patients	34 (36)	53 (56)	8 (8)	0.64	0.36	<0.0001	4.031 (2.211 to 7.350)
Controls	72 (72)	28 (28)	0 (0)	0.86	0.14		
105CT (rs45535938)	CC	CT	TT	C	T		
Patients	88 (93)	6 (6)	1 (1)	0.96	0.04	0.0041	0.282 (0.114 to 0.696)
Controls	78 (78)	21 (21)	1 (1)	0.89	0.11		
541AG (rs2070959)	AA	AG	GG	A	G		
Patients	43 (46)	45 (47)	7 (7)	0.7	0.3	<0.0001	6.853 (3.466 to 13.55)
Controls	85 (85)	14 (14)	1 (1)	0.92	0.08		
552AC (rs1105879)	AA	AC	CC	A	C		
Patients	37 (39)	49 (52)	9 (9)	0.65	0.35	<0.0001	14.11 (6.514 to 30.55)
Controls	90 (90)	8 (8)	2 (2)	0.94	0.06		
IVS1+130GT (rs7592281)	GG	GT	TT	G	T		
Patients	67 (71)	26 (27)	2 (2)	0.85	0.15	0.0053	0.435 (0.241 to 0.785)
Controls	51 (51)	46 (46)	3 (3)	0.74	0.26		

Of the eight genotyped SNPs, five were significantly associated with lung cancer risk, when tested in the first cohort consisting of 72 lung cancer patients and 62 controls. UGT1A6 19T>G, 541A>G and 552A>C were associated with increased lung cancer risk, whereas UGT1A6 105C>T and IVS1+130G>T were inversely associated with lung cancer risk. UGT1A6 19T>G, 541A>G and 552A>C were common in lung cancer patients with minor allele frequencies of 38%, 45% and 48% respectively.

These 5 SNPs were further evaluated in the second cohort consisting of 95 Chinese lung cancer patients and 100 Chinese unmatched controls. UGT1A6 19T>G, 541A>G and 552A>C remained associated with increased lung cancer risk, and UGT1A6 105C>T and IVS1+130G>T remained significantly associated with reduced lung cancer risk.

### Determination of factors associated independently with lung cancer: Univariate and multivariate analyses

To determine factors associated with lung cancer risk, we first used univariate analysis and identified the following factors: age, gender, smoking status, and five SNPs (Table 3). After adjusting for covariables identified by univariate analysis using multivariate analysis we found only smoking status (OR: 3.385, 95% CI: 1.86–6.15, p<0.001), UGT1A6 541A>G (OR: 3.582, 95% CI: 1.27–10.04, p = 0.015), 552A>C (OR: 5.364, 95% CI: 1.92–14.96, p = 0.001) and IVS1+130G>T (OR: 0.191, 95% CI: 0.09–0.36, p<0.001) were independent predictive factor for lung cancer risk.

**Table 3:** Univariate and multivariate models for baseline characteristics.

				Univariate analysis using chi-square test			Multivariate analysis using logistic regression		
Parameter		Patients = 167 (%)	Control = 162 (%)	p-value	OR	95%CI	Adjusted p-value	OR	95%CI
Age (Median)	>63	78 (47%)	1 (0.6%)	<0.001	141	19–1030			
	≤63	89(53%)	161(99.4%)						
Gender	Male	129(77%)	100(62%)	0.002	2.105	1.30–3.40	0.134	1.615	0.86–3.02
	Female	38(23%)	62(38%)						
Smoking	Currentsmoker/ex-smoker	112(67%)	66(41%)	<0.001	2.962	1.89–4.64	<0.001	3.385	1.86–6.15
	Neversmoker	55(33%)	96(59%)						
19TG	TG,GG	111(66%)	50 (31%)	<0.001	4.440	2.79–7.05	0.436	1.331	0.64–2.73
	TT	56(34%)	112(69%)						
105CT	CT,TT	24(14%)	47 (29%)	0.001	0.411	0.23–0.71	0.169	0.602	0.29–1.24
	CC	143(86%)	115 (71%)						
541AG	AG,GG	116(69%)	30(19%)	<0.001	10.008	5.97–16.75	0.015	3.582	1.27–10.04
	AA	51(31%)	132(81%)						
552AC	AC,CC	125(75%)	33(20%)	<0.001	11.634	6.93–19.53	0.001	5.364	1.92–14.96
	AA	42(25%)	129(80%)						
IVS1+130	GT,TT	45(27%)	85(52%)	<0.001	0.334	0.21–0.52	<0.001	0.191	0.09–0.36
	GG	122(73%)	77(48%)						

### Hardy-Weinberg Equilibrium, Linkage Disequilibrium (LD) and Haplotype analysis

Further analysis of the set of DNA polymorphisms that are inherited together, also known as haplotypes was performed with the following results.UGT1A6 19T>G and IVS1+130G>T showed significant deviation from Hardy-Weinberg equilibrium among controls. UGT1A6 552A>C was in close LD with UGT1A6 541A>G (D′ = 0.9706, r^2^ = 0.7795) in lung cancer patients. UGT1A6 105C allele was strongly linked to UGT1A6 541G and 552A allele in control (Table 4). Table 5 summarizes the haplotype frequencies of case-control comparisons from permutation tests. UGT1A6 541A>G were excluded from this analysis because it was in close LD with 552A>C. Ten unique haplotypes could be inferred using 4 risk modifying alleles, with data censored at frequency of 1%.

**Table 4:** Analysis of pairwise linkage disequilibrium (LD) coefficients and statistics (D′) for lung cancer patients (below diagonal) and control (above diagonal) among five SNPs.

	19T>G	105C>T	541A>G	552A>C	IVS1+130G>T
	(rs6759892)	(rs45535938)	(rs2070959)	(rs1105879)	(rs7592281)
19T>G (rs6759892)		−0.1822	0.5688	0.504	0.1226
105C>T (rs45535938)	0.2539		**−0.9999**	**−1**	0.2469
541A>G (rs2070959)	0.7045	0.3647		0.7759	0.5854
552A>C (rs1105879)	0.8419	0.2342	**0.9706**		0.4159
IVS1+130G>T (rs7592281)	0.5757	0.0512	0.3512	0.447	

**Table 5:** Haplotype frequency estimates and significant levels of case-control comparison from permutation tests.

	19T>G	105C>T	552A>C	IVS1+130G>T	Frequency		permutation	OR	CI
	(rs6759892)	(rs45535938)	(rs1105879)	(rs7592281)	Lung cancer	Control	p-value		
1	T	C	A	G	0.466	0.538	ns	0.75	0.55–1.01
2	*G*	C	*C*	G	0.231	0.023	***	13.57	6.15–29.93
3	T	C	A	*T*	0.035	0.142	***	0.23	0.12–0.43
4	T	C	*C*	G	0.077	0.031	**	2.65	1.26–5.59
5	T	*T*	A	G	0.055	0.067	ns	0.78	0.70.41–1.49
6	*G*	C	*C*	*T*	0.096	0.043	*	2.35	1.23–4.48
7	T	*T*	A	*T*	0.008	0.066	***	0.13	0.04–0.44
8	*G*	C	A	G	0.026	0.058	ns	0.44	0.20–1.00
9	*G*	*T*	A	G	0.004	0.018	ns	0.16	0.02–1.33
10	*G*	C	A	*T*	0.004	0.014	ns	0.19	0.02–1.65

* P<0.05,

** P<0.01,

*** P<0.001.

Among these, five haplotypes showed significant p-value in the permutation test, of which, three were associated with increased lung cancer risk. Haplotype #2 containing two risk alleles of UGT1A6 19G and 552C had a higher OR of 13.57 (95% CI: 6.15–29.93) compared to haplotype #4 (OR: 2.65, 95% CI: 1.26–5.59) containing one risk allele of UGT1A6 552A and haplotype #6 (OR: 2.35, 95% CI: 1.23–4.48) containing two risk alleles of UGT1A6 19G and 552C, and one ‘protective’ allele of UGT1A6 intron 1 +130T. Two haplotypes were associated with reduced lung cancer risk, including haplotype #3 (OR: 0.23, 95% CI: 0.12–0.43) and haplotype #7 (OR: 0.13, 95% CI: 0.04–0.44).

### In vitro functional characterization of variant UGT1A6 105TT

**Figure 1:**
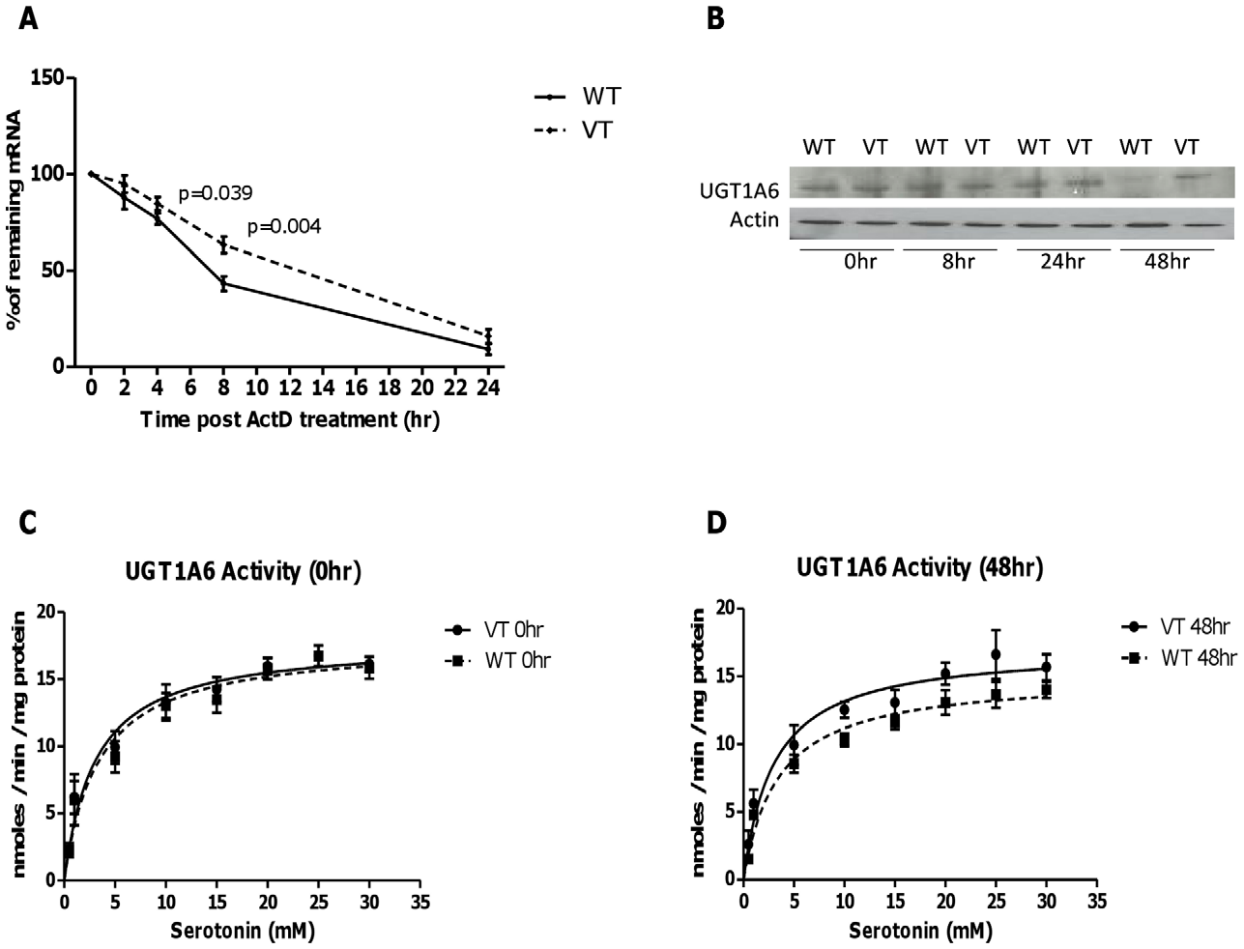
In vitro functional characterization of UGT1A6 105C>T polymorphism. UGT1A6*1 and UGT1A6 105TT constructs were stably transfected into HEK293 cells. (**A**) Cells were harvested upon treatment with ActD at different time point up to 24 hours. The data plotted are time course changes in the remaining amount of UGT1A6 mRNA after ActD treatment. There was significantly higher mRNA level in UGT1A6 105TT than UGT1A6*1 transfected cells at 4 hours (p = 0.039) and 8 hours (p = 0.004) after treatment with ActD. (**B**) Western blot analysis. Cell lysate used was from 5×10^5^ cells at the indicated times (0, 8, 24 and 48 hours) after treatment with ActD. There was a down-regulation in protein expression in UGT1A6*1 as compared to UGT1A6 variant at 48 hours after treatment with ActD. UGT1A6 activity was assessed by evaluating the production of serotonin glucuronide using lysate from variant UGT1A6 (VT) and UGT1A6*1 (WT) at 0 hour (**C**) and 48 hours (**D**) after ActD treatment, with substrate concentrations varied from 0.5 to 30 mM serotonin. Activities are expressed as reaction velocity (nanomoles of serotonin glucuronide formed per minute per milligram of protein).

*In vitro*, mRNA stability was tested after blocking transcription by treating cells with actinomycin D (ActD). Variant UGT1A6 105TT mRNA was more stable than wild type with significantly higher mRNA level observed at 4 hours (p = 0.039), and 8 hours (p = 0.004) after treatment with ActD (Figure 1A). Increased stability of variant UGT1A6 105TT mRNA resulted in higher protein expression and enzyme activity compared to wild-type at 48 hours after treatment with ActD (Figure 1B, C and D).

## Discussion

This is the first study to describe the association between UGT1A6 polymorphisms and lung cancer. The UGT1A6 gene is responsible for the detoxification of carcinogens such as BaP from cigarette smoke [13]–[15]. Quantitative or qualitative alterations in UGT1A6 expression may affect the rate of glucuronidation, thereby modifying the risk of developing lung cancer.

Several polymorphisms have been reported in the 5′-regulatory region, exon 1 and introns of UGT1A6 [24], [25]. Among these, three non-synonymous polymorphisms at codons 7, 181 and 184 (UGT1A6 19T>G, 541A>G and 552A>C, respectively), collectively referred to as UGT1A6*2, was the most commonly studied. It is classified as a ‘low activity’ variant for several phenolic compounds, including aspirin [26] and is associated with reduced colorectal adenoma in subjects taking long-term aspirin [27].

In this study, we had demonstrated that individuals with UGT1A6*2 (haplotype #2 and 6) had increased lung cancer risk. Our finding was consistent with a report of reduced glucuronidation activity in individuals with the UGT1A6*2 allele compared with wild type [26]. Krishnaswamy.*et al* also demonstrated 50% lower UGT1A6 mRNA levels (p = 0.026) in carriers of UGT1A6 19T>G polymorphism compared with noncarriers but without significant effect on UGT1A6 protein content or glucuronidation activities [24]. It should be noted that there were contradictory reports from at least 2 studies, suggesting increased enzyme activity in UGT1A6*2 allozymes [28], [29]. However, the increased activity in the variant UGT1A6 allozyme did not translate into increased substrate clearance in variant human liver microsomes [29]. It is likely that apart from enzyme activity, mRNA degradation may be responsible for the variability of in UGT1A6 activity.

Two polymorphisms UGT1A6 105C>T and IVS1+130G>T were found to be associated with reduced lung cancer risk. The UGT1A6 105C>T is a synonymous SNP that is located in exon 1 of the UGT1A6 gene. Although the polymorphism does not result in qualitative modification of UGT1A6, our *in vitro* results have demonstrated that the T allele is associated with increased mRNA stability and high UGT1A6 activity in variant protein compared to wild-type. This may explain the protective effect of the UGT1A6 105 T allele in reducing lung cancer risk. In addition, the protective effect of UGT1A6 105C>T may be due to its inverse linkage with UGT1A6*2 (UGT1A6 541A>G and 552A>C).

UGT1A6 IVS1+130G>T was found to be the only SNP associated with decreased risk of lung cancer in multivariate analysis. It is unclear how UGT1A6 IVS1+130G>T modulates lung cancer risk as it is located in the first intronic region of the UGT1A6 gene. In-silico analysis using the Human Splicing Finder program (http://www.umd.be/HSF/) suggested that this SNP did not affect any conserved nucleotides of the branch site in the intronic region (data not shown) and would be unlikely to affect splicing activity.

Although we found UGT1A6 IVS1+130G>T to be in moderate linkage disequilibrium with UGT1A6 541A>G (denoted as UGT1A6*5), it cannot explain the protective effect observed. While UGT1A6 is the predominant UGT1A isoform expressed in lung, other UGT1A isoforms (UGT1A1 and 1A9) expressed in the liver may still affect systemic clearance of carcinogens associated with cigarette smoking [30], [31].

It is possible that IVS1+130G>T may be in linkage disequilibrium with polymorphisms in other UGT1A isoforms that play a ‘protective’ role in lung cancer risk. For example, Saeki.*et al* [32] demonstrated that UGT1A6 IVS1+130G>T is in close LD with UGT1A9*22, a high enzymatic activity allele which is known to increase transcription. In addition, UGT1A6 has been demonstrated to be in linkage disequilibrium with UGT1A1 and UGT1A7 [33]–[36]. Although not expressed in lung, these UGT isoforms have been demonstrated to be important in clearance of BaP [30], [31].

The main strength of this study is that our finding was validated in an independent cohort. The analysis was restricted to one ethnic group to minimize the likelihood of false-positive results due to population heterogeneity. There are, however, several limitations in our study that we acknowledge. Firstly, the lung cancer patients and healthy controls were not matched for age, gender and smoking history. Secondly, two SNPs, UGT1A6 19T>G and IVS1+130G>T deviated from Hardy Weinberg equation. Deviation from Hardy Weinberg equation may be caused by genotyping errors [37], [38]. We had taken measure to rule out genotyping error by using both pyrosequencing and direct sequencing method to identify the variant SNPs in our study subjects.

In conclusion, this study suggests that UGT1A6 polymorphisms may modulate lung cancer risk. UGT1A6 105C>T was demonstrated to increase mRNA stability, providing a plausible explanation of its association with reduced lung cancer risk. Our study suggests that UGT1A6 genotyping may be integrated into lung cancer screening strategy to help identify susceptible populations.

## Supporting Information

Table S1 **List of PCR and sequencing primers.** List of all primers used to amplify and sequence each of the eight UGT1A6 SNPs is shown, with information on SNP rsid and their localization provided. The size of PCR products and the annealing temperature for each PCR reaction are also shown. Biotinylated primers for pyrosequencing assay are indicated with §.
(DOC)
