# Supplementary material for: UGT1A6 Polymorphisms Modulated Lung Cancer Risk in a Chinese Population
Source: PLoS One. 2012 Aug 17;7(8):e42873. doi: 10.1371/journal.pone.0042873 (PMC3422233; doi:10.1371/journal.pone.0042873)
Supplement: Table S1 — List of PCR and sequencing primers. List of all primers used to amplify and sequence each of the eight UGT1A6 SNPs is shown, with information on SNP rsid and their localization provided. The size of PCR products and the annealing temperature for each PCR reaction are also shown. Biotinylated primers for pyrosequencing assay are indicated with §. (DOC) [file pone.0042873.s001.doc]

Supplementary Table 1: List of PCR and Sequencing Primers

| SNP | Localization in the gene | Forward and Reverse primers (biotinylated^§^) | Sequencing primer and Pyrosequencing^TM^ Assay type | Annealing Temp (^0^C) | Length of Amplicon(bp) |
| --- | --- | --- | --- | --- | --- |
| –1310del5 (rs45549435) | Promoter | Forward: 5’TAGCAGGCAGGGCCAGTGT3’  Reverse:5’GTACGTGGTGAGAATCCTAGAGCA3’ | Direct sequencing method  Forward assay | 60 | 152 |
| –556C>T (rs45568235) | Promoter | Forward:5’TTGCCAAGTCAGACAGAAGTTGTG3’  Reverse: 5’ATTTTTCTTGCACACGGACCTAT3’^§^ | Sequencing:5’TGGGTAACCTGGGGA3’  Forward assay | 60 | 194 |
| –427G>C (rs12476197) | Promoter | Forward:5’AGTTCAGAATGGAATTGAATTGTA3’  Reverse: 5’CTTGCTGGATTCCTCATCATT3’^§^ | Sequencing: 5’ATACCCAGCTGGTGTT3’  Forward assay | 55 | 123 |
| 19T>G (rs6759892) | Exon 1 | Forward: 5’GATGGCCTGCCTCCTTCG3’  Reverse: 5’GGACCACCAGCAGCTTGTCA3’^§^ | Sequencing: 5’GCCTGCCTCCTTCGC3’  Forward assay | 55 | 98 |
| 105C>T (rs45535938) | Exon 1 | Forward: 5’TGCAGGGGTTTTCTTCTTAGCA3’  Reverse:5’AACTTCAGGCACCACCACTAC3’^§^ | Sequencing: 5’GCTGGTGGTCCCTCA3’  Forward assay | 55 | 154 |
| 541A>G (rs2070959) | Exon 1 | Forward: 5’TACCTCTTCAGGGGTTTTCCG3’  Reverse:5’TTAACAAGGAAGTTGGCCACTC3’^§^ | Sequencing: 5’CCGTGTTCCCTGGAG3’  Forward assay | 55 | 140 |
| 552A>C (rs1105879) | Exon 1 | Forward: 5’TACCTCTTCAGGGGTTTTCCG3’  Reverse:5’TTAACAAGGAAGTTGGCCACTC3’^§^ | Sequencing: 5’CCGTGTTCCCTGGAG3’  Forward assay | 55 | 140 |
| 130G>T (rs7592281) | Intron 1 (IVS1) | Forward:5’TGGAGGATTTCCTGGAGAAACG3’  Reverse: 5’CCCTGTCTTCATCCGAATGC3’^§^ | Sequencing: 5’GGGGGAAGTGATACC3’  Forward assay | 55 | 113 |
